# COMP Report: CPQR technical quality control guidelines for use of positron emission tomography/computed tomography in radiation treatment planning

**DOI:** 10.1002/acm2.13785

**Published:** 2022-10-08

**Authors:** Ran Klein, Mike Oliver, Dan La Russa, John Agapito, Stewart Gaede, JeanPierre Bissonnette, Arman Rahmim, Carlos Uribe

**Affiliations:** ^1^ Department of Nuclear Medicine The Ottawa Hospital Ottawa Canada; ^2^ Health Sciences North Sudbury Canada; ^3^ Radiation Medicine Program The Ottawa Hospital Canada; ^4^ Department of Medical Physics Windsor Regional Hospital Windsor Canada; ^5^ London Regional Cancer Program London Health Sciences Centre London Canada; ^6^ Department of Medical Physics Princess Margaret Cancer Centre Toronto Canada; ^7^ Functional Imaging BC Cancer Agency Vancouver Canada

**Keywords:** PET, PET/CT, quality control, radiation therapy, treatment planning

## Abstract

Positron emission tomography with x‐ray computed tomography (PET/CT) is increasingly being utilized for radiation treatment planning (RTP). Accurate delivery of RT therefore depends on quality PET/CT data. This study covers quality control (QC) procedures required for PET/CT for diagnostic imaging and incremental QC required for RTP. Based on a review of the literature, it compiles a list of recommended tests, performance frequencies, and tolerances, as well as references to documents detailing how to perform each test. The report was commissioned by the Canadian Organization of Medical Physicists as part of the Canadian Partnership for Quality Radiotherapy initiative.

## 1 INTRODUCTION

The Canadian Partnership for Quality Radiotherapy (CPQR) is an alliance among the three key national professional organizations involved in the delivery of radiation treatment in Canada: the Canadian Association of Radiation Oncology (CARO), the Canadian Organization of Medical Physicists (COMP), and the Canadian Association of Medical Radiation Technologists (CAMRT), together with financial and strategic backing from the Canadian Partnership Against Cancer (CPAC) which works with Canada's cancer community to reduce the burden of cancer on Canadians. The vision and mandate of the CPQR is to support the universal availability of high‐quality and safe radiotherapy for all Canadians through system performance improvement, and the development of consensus‐based guidelines and indicators to aid in radiation treatment program development and evaluation.

This report contains detailed performance objectives and safety criteria for positron emission tomography for radiation/computed tomography treatment planning (PET/CT for RTP). Please refer to the overarching document *Technical Quality Control Guidelines for Canadian Radiation Treatment Centres* ^1^, ^2^ for a programmatic overview of technical quality control (TQC), and a description of how the performance objectives and criteria listed in this document should be interpreted. TQC documents are live and may be updated from time to time. To avoid duplication and risk of conflicting information, other TQC documents are referred to, but not reproduced. Tests that are not covered in other TQC documents are detailed herein. A detailed description of the production, review, and impact of TQC guidelines was previously described in this journal.^3^

In the RTP process, physiological information from positron emission tomography (PET) can be used to inform target delineation and identify metabolically active regions for possible dose escalation. Information from PET scans can also be used to help spare healthy tissue, further boosting the probability of a complication‐free cure. PET‐based RTP, however, is a relatively new application that is not yet commonly utilized and requires quality control (QC) measures that are incremental to that of routine diagnostic PET. This report reviews current QC guidelines for combined PET and x‐ray CT for RTP to produce a consolidated list of QC tests for PET‐based RTP. These incremental QC activities are relatively few and should not pose a major obstacle for expending the use of PET to RTP.

This report forms a review and summary of existing guidelines and recommendations relevant to QC of PET for RTP. It was assembled by a committee of subject matter experts as a guideline for practitioners looking to implement PET for RTP. The report was reviewed and vetted through the CPQR TQC process. This publication is an adaptation of the original TQC^4^ and intends to:

1. Recommend QC procedures of PET/CT for RTP in the absence of prior works dedicated to this emerging application.

2. Make readers aware of CPQR TQC documents in general as a resource for establishing stringent QC in radiation treatment.

## 2 SYSTEM DESCRIPTION

Radiation therapy aims to accurately deposit a prescribed amount of radiation dose to target volumes, while sparing surrounding disease‐free tissues. To achieve this goal, the radiological properties of the patient anatomy must be accurately represented in the treatment planning system for dose‐calculation purposes. This anatomical information, along with delineated target and avoidance structures, is routinely derived from CT‐simulator images.

Modern, hybrid PET/CT system combines a PET sub‐system to generate 3D images of functional processes in the body and a co‐registered CT sub‐system. The CT generates attenuation images for anatomical lesion localization while allowing for accurate photon attenuation correction of the PET images. These hybrid systems are often equipped with fully diagnostic CT scanners that can also serve as CT simulators for RTP. With the addition of a flat tabletop and isocenter lasers, these hybrid systems could well fulfil the requirements for CT simulation. Four perceived methods of PET/CT for RTP can be envisioned in order of increasing technical complexity and treatment accuracy:

1. Side‐by‐side visualization of the diagnostic PET/CT data and a second CT‐simulator image, whereby the radiation oncologist manually defines the treatment volumes on the CT‐simulator image using the PET/CT for guidance, as accurate image registration between the different patient postures may be challenging.^5^ This method relies on existing practices and does not leverage the full power of modern PET/CT for RTP and simulation. It is limited by low operator reproducibility and accuracy.
2. Software‐based registration of the PET/CT study with the CT‐simulator image to guide the definition of treatment volumes.^6^ In theory, this approach overcomes the above limitations and provides optimal registration between the PET and the simulation CT images. Registration between images is usually achieved by affine registration between the two CTs, and is aided by consistent patient positioning in the RT posture. While deformable, non‐rigid image registration that can compensate for inconsistent patient positioning is an ongoing topic of research, routine clinical application is not yet widely feasible. Thus, inaccurate image registration limits the accuracy of target delineation and subsequent treatment planning.
3. Acquisition of the PET/CT data with the patient in the RTP configuration and using these data for target volume delineation and planning without the need for an additional CT‐simulator image. This method aims to fully exploit the information in PET/CT both for target delineation and RTP dose calculations, but also requires a flat tabletop and a wall‐mounted laser alignment system be installed in the PET/CT imaging suite to accurately register the patient in the treatment planning system and RT treatment machine. To date, widespread adoption of PET/CT simulators has been limited by workflow constraints and lack of reimbursement. Nevertheless, this method is proposed as a feasible option due to the recent trend toward clinical utilization of PET/CT simulators, the decreased cost in fluorodeoxyglucose (FDG), and because the QC testing required for this method encompasses the requirements for methods 1 and 2 above. It should be appreciated, however, that incorporating the entire RT simulation process into the PET/CT image acquisition workflow, which often includes the design and application of immobilization devices and patient indexing, can result in prolonged PET/CT appointment times. This will undoubtedly reduce patient throughput on PET imaging systems and risk increases to staff exposures.^7^
4. A viable alternative to combining the RT simulation and PET/CT image acquisition processes is to first perform RTP on a CT simulator and then replicate patient positioning in the PET/CT, enabling accurate image registration through simple rigid transformations. These procedures should include steps for converting the PET/CT system to accommodate a flat tabletop and patient immobilization device that can be rapidly, consistently, and safely deployed. Special considerations should be given to potentially smaller bore sizes of PET/CT systems that may limit patient positioning. This approach to acquiring PET/CT images will facilitate accurate image registration for treatment planning CT images.^8^, ^9^, ^10^

QA of PET/CT simulators is largely similar to the QA of CT simulators, with the addition of PET‐dedicated QC tests. Since rigorous TQC guidelines for CT simulators have already been established by CPQR^11^ and are actively being maintained, supplementary guidelines should be created for PET/CT with minimal duplication to avoid inconsistencies, as these guidelines evolve over time. PET/CT devices are rarely dedicated to RT and therefore may reside in the diagnostic imaging department (e.g., nuclear medicine or radiology). Sharing of responsibilities between departments and close coordination is essential to ensure quality of the overall PET/CT‐simulator process.

## 3 RELATED TECHNICAL QUALITY CONTROL GUIDELINES

### 3.1 Performance testing

Performance tests should be referred to when selecting a system, when performing acceptance evaluation of newly installed equipment, and prior to the end of a manufacturer's warranty period. The National Electrical Measurements Association (NEMA) has developed standard NU‐2‐2012,^12^ which has become the de facto standard for evaluating the performance of PET systems. The standard describes equipment and procedures for measuring system performance parameters including spatial resolution, scatter fraction, count losses, random events measurement, activity sensitivity, corrections accuracy, and image quality. The NEMA standard was updated to version NU‐2‐2018,^13^ adding two new procedures to assess coincidence timing resolution on PET systems with time‐of‐flight capability, and to assess co‐registration accuracy of hybrid PET/CT systems; the latter is of particular interest in using PET for RTP. Task Group 126 on the American Association of Physicists in Medicine (AAPM) has put out a report entitled PET/CT Acceptance Testing and Quality Assurance that recommends an alternative set of simplified tests that may be performed with fewer specialized phantoms and equipment.^14^ Likewise, performance testing of CT equipment are detailed by the AAPM,^15^ International Electrotechnical Commission (IEC),^16^ and other similar professional body recommendation documents. These tests are also summarized in an IAEA document.^17^

### 3.2 Acceptance testing and commissioning

Newly acquired or substantially modified PET/CT systems should be tested to ensure performance complies with vendor‐ and tender‐stated specifications.^18^, ^19^ Through active participation in the acceptance testing, users may also become familiar with the system. Commissioning follows acceptance testing with a comprehensive battery of performance tests to establish base‐line performance metrics against which subsequent tests may be compared to ensure stable and acceptable performance of the system over its lifetime.

### 3.3 System upgrades and maintenance

Special consideration should be given in the case of a PET/CT system servicing and upgrades. Acceptance or preventive maintenance tests provided by the PET/CT manufacturer under an institutional service contract agreement should ensure that the PET/CT system is at optimal functionality. However, monthly tests should be performed after any hardware upgrade and both monthly and annual QC should be done after PET/CT console software upgrade.

### 3.4 Routine QC

Routine QC is performed to ensure system stability from time of commissioning and to proactively determine the need for service. Periodic (e.g., daily, monthly, quarterly) QC tests are typically defined by the manufacturer and may differ from general guidelines due to technology (e.g., solid‐state vs. photomultiplier tube‐based detection) and feasibility considerations (e.g., automated QC). Routine QC guidelines have been established by multiple professional groups with a consensus statement on Diagnostic Imaging Requirements put out by the Joint Commission on the Accreditation of Healthcare Organizations as an umbrella list of QA requirements.^20^ The CPQR has established its own TQC guidelines as summary standards of test frequency and tolerances.^3^

Recommendations from major international professional bodies (listed in Table 1) were included in this review. A summary of recommended routine QC activities and frequencies is summarized in the Test Tables section along with references in which greater details on the QC test may be found. Tolerances from TQCs were used if available, otherwise the strictest values from the reviewed literature were adopted. We elected to recommend the strictest tolerances to accommodate the use of PET/CT acquired explicitly for RTP (mode 3 above) which is most demanding of quality. The list is intended to serve as a guideline and may not be optimal for all equipment types and all applications (e.g., modes 1, 2, and 4); in which case adaptations to the test and/or relaxation of tolerances may be made in consultation with a knowledgeable medical physicist. For comprehensive instructions for performing PET/CT QA, the reader is referred to references.^17^, ^21^

**TABLE 1:** List of related quality control references reviewed

**Title**	**Revision year**	**Professional Body**	**Modality**	**Reference**
Task Group 126 Report: PET/CT acceptance testing and quality assurance	2019	American Association of Physicists in Medicine	PET‐CT	^14^
Task Group 174 Report: Utilization of [^18^F]fluorodeoxyglucose positron emission tomography ([^18^F]FDG‐PET in radiation therapy	2019	American Association of Physicists in Medicine	PET‐CT	^10^
Technical quality control guidelines for computed tomography simulators	2016	Canadian Partnership for Quality Radiotherapy	CT simulator	^11^
Technical standard for medical nuclear physics performance monitoring of PET/CT imaging equipment	2018	American College of Radiologists & American Association of Physicists in Medicine	PET‐CT	^21^
Diagnostic imaging requirements	2015	Joint Commission on the Accreditation of Healthcare Organizations	PET, CT, MRI, NM	^20^
Routine quality control recommendations for nuclear medicine instrumentation	2010	European Association of Nuclear Medicine	PET, dose calibrator	^24^
PET/CT and radiotherapy: data transfer, radiotherapy workflow and quality assurance	2010	‐	PET, CT, RTP	^5^
Quality assurance for PET and PET/CT systems	2009	International Atomic Energy Agency	PET, CT	^17^
Quality assurance of PET/CT for radiation therapy	2008	‐	PET, CT, RTP	^23^
Routine quality control of clinical nuclear medicine instrumentation: A brief review	2008	‐	PET, CT, dose calibrator	^36^
Quality assurance for computed‐tomography simulators and the computed‐tomography‐simulation process: Report of the AAPM Radiation Therapy Committee Task Group N. 66	2003	American Association of Physicists in Medicine	CT simulator	^28^

In order to comprehensively assess the use of PET/CT for RTP performance, additional tests, as outlined in related CPQR TQC guidelines must also be completed and documented, as applicable. Related TQC guidelines, available at https://www.cpqr.ca/programs/technical‐quality‐control/ (and a subset have been published through the journal), include:

- Treatment planning systems
- Computed tomography simulators^22^
- Data management systems

TQC guidelines are referred to throughout as a primary source to avoid conflicting instructions as these live documents are updated.

The QA program should be overseen by trained medical physicist(s) with expertise in diagnostic imaging, nuclear medicine, and RT. Frequent QC tests may be delegated to trained technologists, but results should be reviewed by a physicist in a timely manner to identify equipment that does not meet operating specifications.

## 4 TEST TABLES

Tables 2, 3, 4, 5 to 6 list required PET/CT for RTP QC tests by frequency. These tables further indicate incremental tests over those typically required for diagnostic PET/CT as derived from the CPQR TQC guidelines for CT simulators^11^ (✓ = incremental tests, 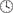 = tests performed more frequently).

**TABLE 2:** Daily/weekly quality control tests

**^Designator^**		**^Tolerance^**	**^For RTP^**
PT‐D1	PET detector stability	Manufacturer's recommendation	
PT‐D2	Daily coincidence timing resolution tests in TOF PETs	Manufacturer's recommendation	
CT‐D1	External lasers (alignment, spacing, motion)^a^	±1 mm	✓
CT‐D2	CT number for water–mean (accuracy)^a,b^	0±4 HU	
CT‐D3	CT number for water–standard deviation (noise)^a,b^	Reproducible (±10% or 0.2 HU from baseline value, whichever is larger)	
CT‐D4	CT number for water–mean vs. position (uniformity)^a^	±2 HU	
CT‐D5	Respiratory monitoring system	Functional	
CT‐D6	Audio/video coaching systems (if applicable)	Functional	
PT‐W1	Adjustment of gains of photomultiplier tubes	Manufacturer's recommendation	
OT‐D1	Dose calibrator accuracy (clock accuracy, high voltage, zero adjustment, background activity, constancy)	±5%	

Daily

^a^ Can be performed weekly if system is found to be stable but needed on days system will be used for RTP.

^b^ Alternate to cover all peak kilovoltage (kVp) values used clinically.

**TABLE 3:** Monthly quality control tests

**Designator**		**Tolerance**	**For RTP**
CT‐M1	Table top level accuracy^a^	±2 mm	✓
CT‐M2	External lasers (orthogonality/orientation)	± 1 mm over the length of laser projection	✓
CT‐M3	Table top displacement accuracy^a^	±1 mm	✓
G‐M1	Records	Complete	

Monthly (or after system maintenance)

^a^ Perform whenever table top is removed and reinstalled.

**TABLE 4:** Quarterly quality control tests

**Designator**		**Tolerance**	**For RTP**
Quarterly (or after system maintenance)			
PT‐Q1	PET System normalization and calibration	Visual acceptance. The new calibration should be checked with a reconstructed image of the flood phantom applying all the corrections. The mean measured SUV in a region of 10 cm in the center of the phantom should be 1.0 ± 0.1. Calibration constant change <5% from previous.^25^, ^26^	
PT‐Q2	Uniformity of reconstructed PET image	Within 5% of baseline value	
PT‐Q3	PET and CT registration	±1 PET pixel (or ±1 mm if PET pixel is <1 mm)	
CT‐Q1	CT number accuracy (>4 materials)	±5 HU	
CT‐Q2	3D low contrast resolution	Reproducible (set action level at time of acceptance)	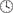
CT‐Q3	3D high contrast spatial resolution (at 10 and 50% modulation transfer function [MTF])	Reproducible (±0.5 lp/cm or ±15% of the established baseline value, whichever is greater)	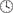
CT‐Q4	Slice thickness (sensitivity profile)	Reproducible (±0.5 mm from baseline for slices ≥2 mm ±50% from baseline for slices of 1 to 2 mm ±0.5 mm from baseline for slices <1 mm)	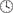
CT‐Q5	Amplitude and periodicity of motion surrogate with monitoring software and/or CT console	1 mm, 0.1 s	✓
CT‐Q6	4D‐CT reconstruction	Functional	✓
CT‐Q7	Amplitude of moving target(s) measured with 4D‐CT	<2 mm	✓
CT‐Q8	Spatial integrity and positioning of moving target(s) at each 4D respiratory phase	2 mm (FWHM) difference from baseline measurement (increased for amplitudes larger than 2 cm)	✓
CT‐Q9	Mean CT number and standard deviation of moving target(s) at each respiratory phase	(±10 HU) and (±10%) from baseline measurement (increased for amplitudes larger than 2 cm)	✓
CT‐Q10	4D‐CT intensity projection image reconstruction (Avg, MIP, MinIP)	2 mm (FWHM) difference from baseline measurement (increased for amplitudes larger than 2 cm)	✓
CT‐Q11	4D data import to treatment planning system	Functional	✓
OT‐Q1	Dose calibrator linearity	Manufacturer's recommendation	

**TABLE 5:** Annual quality control tests

**Designator**		**Tolerance**	**For RTP**
**Annually**			
PT‐A1	Safety: mechanical and electrical	Manufacturer's recommendation	
PT‐A2	PET spatial resolution	Manufacturer's Specifications	
PT‐A3	PET sensitivity	Manufacturer's Specifications	
PT‐A4	PET image quality phantom (hot spheres, cold rods, quantitative accuracy)	Baseline CT and PET spatial integrity: phantom width and height dimension errors ≤2 pixel (or ≤2 mm).	
PT‐A5	PET count rate performance (scatter fraction, count losses, randoms)	Manufacturer's Specifications	
PT‐A6	Time‐of‐flight resolution (if applicable)	Baseline	
CT‐A1	Patient dose from CT, CTDI (or x‐ray source radiation profile width); adult and pediatric	±10% from baseline	
CT‐A2	X‐ray generation: kVp, HVL, mAs linearity	±2 kVp, ±10% difference from baseline measurement (HVL and mAs)	
CT‐A3	Gantry tilt (if applicable)	±0.5%	✓
CT‐A4	4D low contrast resolution at each respiratory phase	Reproducible (set action level at time of acceptance)	
CT‐A5	4D high contrast spatial resolution at each respiratory phase	Reproducible (set action level at time of acceptance)	
CT‐A6	4D slice thickness (sensitivity profile) at each respiratory phase	Reproducible (set action level at time of acceptance)	
CT‐A7	Simulated planning	±2 mm	
G‐A1	Records	Complete	
G‐A2	Independent quality control review	Complete	
G‐A3	Review of long‐term trends for quantitative daily and monthly tests	Complete	
Other			
OT‐A1	Dose calibrator geometry accuracy	5%	
OT‐A2	Computer monitor display accuracy	Manufacturer's recommendation	
OT‐A3	Patient weight scale accuracy and precision	±0.1 kg for weights <100 kg ±0.2 kg for weights ≥100 kg	
OT‐A4	Patient height measurement device	±5 mm	

**TABLE 6:** Patient‐specific quality control tests

**Designator**		**Tolerance**	**For RTP**
**Case‐by‐Case**			
PS1	Correct patient	Matched patient identifying information	
PS2	Correct patient preparation	Matched to requisition	
PS3	Correct patient positioning	Matched to treatment plan	
PS4	Correct imaging protocol and parameters	Matched to requisition and technologist's worksheet	
PS5	PET/CT Image registration	Adequate co‐registration	
PS6	Image quality	Diagnostic image quality	

### Notes on daily/weekly tests (Table 2)

**Table:** 

PT‐D1 ‐ 2 and PT‐W1	As per manufacturer's instructions, these tests are typically semiautomated and only require confirmation that the test has passed, and no visual artifacts are visible in the recorded sinograms. The tests measure the stability of the PET detectors.
	On scanners with TOF, PT‐D2 measures the capability of the system to estimate the difference in arrival times of the two annihilation photons.
	The weekly test updates the detector gains to compensate for changes in the crystals’ behavior over time. See references^17^, ^23^ for more details.
CT‐D1 ‐ 6	Refer to the TQC for computed tomography simulators.^11^
OT‐D1	As per manufacturer instructions, follow the daily QC procedure using a long‐lived radionuclide source (e.g., ^137^Cs) to test accuracy and stability.^24^

### Notes on monthly tests (Table 3)

**Table:** 

CT‐M1 ‐ 3	Refer to TQC for computed tomography simulators.^11^
G‐M1	Documentation relating to the daily QC checks, preventive maintenance, service calls, and subsequent checks must be complete, legible, and the operator identified.^19^

### Notes on quarterly tests (Table 4)

**Table:** 

PT‐Q1	This test measures the crystal efficiency and is used to correct for crystal non‐uniformities that degrade the images. The scanner is also cross‐calibrated with the dose calibrator to ensure that SUV calculations are accurate, and the images are quantitative.^17^
	The test is performed using a cylindrical uniform phantom of known activity concentration (depending on the manufacturer's recommendations it can be a pre‐manufactured ^68^Ge phantom or a fillable one with ^18^F). The normalization data are acquired according to the manufacturer's instructions. A calibration factor relating the detected events to the known activity concentration is also calculated. The test passes when a reconstructed image using the new established normalization and calibration factor parameters is visually uniform, and the measured SUV_mean_ in a big field of view inside the phantom is close to 1. Data should also be compared with previous measurements to detect big shifts in calibration, which could indicate procedural errors in the test.^25^, ^26^
PT‐Q2	The cylindrical phantom from the PT‐Q1 test is used to measure the response of the system to the homogeneous activity distribution.^17^ An image of the phantom is reconstructed with all the corrections enabled (i.e., dead time, attenuation, scatter, etc.) and using the parameters of the institution's standard clinical protocol. For each transaxial slice in the image, a grid of 10 mm × 10 mm squares is drawn. The maximum, minimum, and mean concentration *c* of each grid element *k* in each of the *i* image slices is recorded. The maximum value of non‐uniformity across all images (*NU_i_*) should be reported as per equation 1 (1) $$NU_{i}=MAX\left\{\begin{array}{c} \frac{MAX\left(c_{k}\right)-AVE\left(c_{k}\right)}{AVE\left(c_{k}\right)}\times 100\\ \frac{AVE\left(c_{k}\right)-MIN\left(c_{k}\right)}{AVE\left(c_{k}\right)}\times 100 \end{array}\right.$$
PT‐Q3	This test ensures that the functional information of PET is correctly aligned with the anatomical information from CT.^17^ The alignment depends on the mechanical components of the PET/CT scanner. Additional corrections are made via a software calculated transformation matrix that translates and rotates one image domain to the other one. The procedure varies based on the manufacturer. Typically, a phantom containing small ^68^Ge sources placed at different positions within the FOV is scanned both on CT and PET. Weights may be added on top of the bed to simulate the effect of having a patient on top of it. The transformation matrix is calculated such that the centroids of the different sources in both scanning modalities are registered.
	The new NEMA NU 2–2018 document^13^ has added a new standard for PET/CT co‐registration. It uses fiducial markers of sources such as ^18^F or ^22^Na with materials that are greater than 500 Hounsfield units in the CT scan. The location of the centroids in the two images are checked to determine the co‐registration error, CE, for each of the fiducial markers using equation 2.
$\mathrm{CE}=\sqrt{{\left({x_{\mathrm{cent}}}_{\mathrm{PET}}-{x_{\mathrm{cent}}}_{\mathrm{CT}}\right)}^{2}+{\left({y_{\mathrm{cent}}}_{\mathrm{PET}}-{y_{\mathrm{cent}}}_{\mathrm{CT}}\right)}^{2}+{\left({z_{\mathrm{cent}}}_{\mathrm{PET}}-{z_{\mathrm{cent}}}_{\mathrm{CT}}\right)}^{2}}$	
	As this report is still recent, not all current PET/CT scanners follow this exact procedure. Regardless, patient‐specific bed sag and misregistration may not be fully characterized by QC, and successful QC may not preclude the need for patient‐specific compensation (e.g., using fiducial markers).
CT‐Q1 ‐ 11	Refer to the TQC for computed tomography simulators.^11^
OT‐Q1	The linearity test measures the response to radionuclides over a big range of activities that will be used in the department. A vial containing a high amount of activity is measured several times until the activity has decayed to a low value. We recommend performing the test with a starting activity on the order of a few GBq and decay until the activity is less than 1 MBq. The measured activity in the dose calibrator is compared to the predicted activity based on the half‐life of the decay. This response is expected to follow the identity line in a plot of measured activity vs. predicted activity. Alternatively, specially designed attenuation sleeves (calibrated for the test isotope) can be used as a surrogate for activity decay.
	Details may be found in reference.^24^

### Notes on annual tests (Table 5)

**Table:** 

PT‐A1	This test ensures that the PET/CT scanner mechanical and electrical components are operating as indicated by the manufacturer. Follow any manufacturer's recommendations and inspect the housing, bed motion, controls, connectors, and any accessories that are connected to the scanner.^17^, ^21^, ^27^
PT‐A2	The aim of this test is to measure the tomographic resolution in air and ensure that is not affected by the acquisition or reconstruction. The procedure involves scanning three‐point sources of ^18^F that are prepared from a high activity concentration in capillary tubes. The tubes are placed in three different positions within the FOV but are always in the same longitudinal plane. The positioning of the sources within the FOV has been updated between different versions of the NEMA standards so it is important to check with the manufacturer to determine which version of the standards should be followed. The acquired images are reconstructed with a pixel size of 1/3 of the expected scanner resolution (typically <1.5 mm per pixel). Profiles of the sources are generated in all the different directions. The full width at half maximum (FWHM) and full width at tenth of maximum (FWTM) are calculated. The radial and tangential resolutions are averaged. The FWHM should not exceed the specifications provided by the manufacturer.^13^, ^17^, ^21^
PT‐A3	This test determines the rate of detected true coincidences per unit of radioactivity concentration (e.g., kcps/MBq) for a standard line source configuration. Several scans of a line source with different aluminum sleeves that increase the thickness of absorbing material are used to extrapolate the value to the one where no attenuating material is present. The procedure is performed at the center of the FOV and at 10 cm from the central axis. The sensitivity is expected to be equal or greater than the specified by the scanner manufacturer.^13^, ^17^, ^21^
PT‐A4	The purpose of this test is to generate images that simulate a real patient scan with hot and cold lesions and with scatter from outside of the FOV. The quality of the image is assessed from the contrast and background variability, accuracy of the attenuation and scatter corrections, and from the accuracy of the radioactivity quantification. The procedure involves scanning the NEMA IEC body phantom that includes six spheres of different sizes.
	The two biggest spheres are filled with water that does not contain radioactivity; while the other four are filled with a solution that has a concentration of 8 times the one in the background (some manufacturers also suggest using a 4:1 ratio). A line source is placed inside a cylindrical plastic phantom to generate some scatter out of the FOV. The images should be reconstructed as recommended by the manufacturer for a standard whole‐body protocol.
	The slice in which the contrast of cold and hot spheres is highest is selected to draw regions of interest around each of the spheres. The diameters of the region of interests (ROIs) should be as close to the inner diameter of the sphere as possible. Concentric ROIs of the same sizes (both cold and hot spheres) are drawn on the same slice at 12 background regions (see NEMA standards for location of ROIs). The same ROIs are then copied to four neighboring slices (∼±1 and ±2 cm) giving a total of 60 background ROIs for each size of sphere; 12 on each of the 5 slices. The average number of counts in each hot, background, and cold spheres in combination with the known activity concentrations are used to calculate the contrast and background variability.
	An ROI with a diameter of 3.0 cm is drawn on the lung insert for each of the slices. If the scatter and attenuation correction are perfect, this value is expected to be close to zero. Another 12 circular 3.0 cm diameter ROIs placed over the background region are used to calculate a percentage relative error for the lung insert and for each slice. The ratio of the average counts in the lung ROI to the average in the 12 background ROIs in the corresponding slice is calculated as a percentage.
	Lastly, accuracy in activity quantification is measured from the known activity in the background at the time of the phantom filling procedure and comparing it to the average radioactivity concentration measured from the image by averaging the 12 3.7 cm diameter background ROIs.
	Using a fused‐image display, ensure accurate registration between PET and CT images. Measure the width and height of the phantom shell on the CT image and ensure that they agree with the physical measurements within 2 pixel width (or 2 mm) to ensure spatial integrity of both PET and CT modalities.
	See references^13^, ^17^, ^21^ for further details.
PT‐A5	This test measures the contribution of scatter, count losses, and random to the image. All of these effects degrade the image quality and quantification accuracy. A small scatter fraction (ratio of scatter photons to the sum of true coincidences and scatter) is desired. The count rate performance provides information regarding the quantitative accuracy at low and high count rates. The noise equivalent count rate (NECR) is typically used to represent the count rate performance as a function of the activity concentration. The peak NECR and the corresponding activity concentration serve as a guide to optimize the injected activity to patients. The calculation assumes Poisson statistics, and considers the contribution of true, scattered, and random events to the total coincidence rate.
	The method of measurement involves a 70‐cm long line source that is placed inside and off‐center of a plastic cylinder. The manufacturer's specifications for the initial radioactivity concentration within the line source should be followed. Different acquisitions are taken at intervals of less than half of the half‐life of the radioisotope (e.g., ^18^F), but with a higher frequency around the peak of the NECR curve. Each acquisition has a duration that should be less than ¼ of the half‐life of the radioisotope. The analysis might be slightly different between systems depending on the method of random correction.
	Pixels that are more than 12 cm away from the center of each sinogram (i.e., one sinogram per acquisition) are set to zero. Then, the maximum pixel on each projection (row) of the sinogram is shifted to the center of the sinogram and all the projections are added. A profile of total counts as a function of distance from the center of the sinogram is made. The sum of scatter and random, the total counts, and the unscattered counts can be determined from that profile. The scatter fraction is then calculated for each slice and each acquisition. The NECR for each acquisition *j* is calculated based on the trues, total, and randoms of each slice *i* using equation 3. (3) $NECR_{i,j}=\frac{R_{t_{i}{,}_{j}}^{2}}{\left(R_{tot}{{}_{i}}_{,j}+\kappa {R_{r}}_{i,j}\right)}$
	where $R_{t}$ is the rate of true coincidences, $R_{tot}$ is the total count rate, and $R_{r}$ represents the randoms count rate. The value of κ is set to 0 for processed delayed window or singles event rates (noiseless), or to 1 for unprocessed delayed window (noisy) randoms correction mode.
	The total system NECR for an acquisition *j* is the sum of $NECR_{i,j}$ over all the slices *i*.
	The scatter fraction, peak NECR, and the radioactivity concentration to reach the peak NECR should meet the manufacturer's specifications.
	See references^13^, ^17^, ^21^ for further details.
PT‐A6	This test determines the capability of the system to measure the difference in arrival time of two coincidence events. Follow the manufacturer's recommendations to perform this test. A typical measurement uses a line source of ^18^F in an aluminum tube positioned at the center of the scanner. The system records coincidences with time of arrival and generates some histograms for it. The timing resolution is calculated as the FWHM on this histogram. The timing resolution should not exceed the manufacturer's specifications.^13^, ^17^, ^21^
CT‐A1 ‐ 7	Refer to the TQC for computed tomography simulators.^11^, ^28^
GA1 – 3	Refer to the TQC for computed tomography simulators.^11^, ^19^
OT‐A1	The dose calibrator geometry test allows determining if the correct activity values are measured regardless of the sample size geometry. For this, all the different syringes and vials used to draw‐up injected doses are tested. For each of the volumes, an initial value of activity is measured. This is then followed by subsequent measurements in which a saline solution or water is added to the syringe/vial to increase the volume. In all cases, the activity is expected to be within 5% of the initial values. If variations >±5% exist, derive a calibration factor to be applied clinically. Ensure no change from baseline. Likewise, using a syringe test stability of the activity reading as the source is gradually withdrawn from the ionization chamber. Ensure that activity readings are consistent across >5 cm of displacement, and that response is consistent with baseline.
	Details may be found in reference.^24^
OT‐A2	Clinical computer monitor displays should be tested and calibrated at least annually using a dedicated light measurement device and according to its manufacturer procedure. As a minimum, displays that have obvious discoloring, non‐uniform luminance >30%, or that deviate from DICOM luminance response accuracy by >10% and cannot be calibrated should be replaced.^29^
OT‐A3	No regulatory guidelines or standards could be found for QC of medical weight scales. But vendor provided instructions require testing using standard weights on the order of typical patient weights (e.g., 100 kg). Testing should be performed on an annual basis, after relocating the device or after service. Errors should not exceed 0.1 kg for weights <100 kg of 0.2 kg for larger weights.
OT‐A4	No guidelines or standards could be found for QC of height measurement devices. Accuracy should be tested annually, after relocating or after service using an independent measuring device such as a measuring tape.

### Notes on patient‐specific tests (Table 6)

At least three forms should be filled to ensure that the PET/CT procedure is going to be performed optimally:

1. A screening form should be filled by the booking clerk ensuring that contains information regarding patient medication, diabetes, claustrophobia, concerns lying flat for the PET/CT scan, and for females, whether they are pregnant or breastfeeding.
2. A questionnaire form to be filled by the patient and to be presented on the day of the appointment. This form should include some questions regarding the patient's clinical history (e.g., asthmatic, diabetic, smoke status). It should contain information about any implants or other foreign objects within the patient's body. In addition, it should include a small questionnaire in the type of “checkboxes” to ensure that the patient has fasted before the appointment (if required), is well hydrated, and has listed his current medications.
3. A PET/CT technologist's worksheet that includes patient information such as name, date of birth, and age. The technologists should record the patient's weight and height, glucose level, allergies, radioisotope to be administered, and should record the initial activity in the syringe, and the residual after injection with its respective times of measurement. Additionally, the volume of radiotracer and the site of injection should also be recorded. The scan protocol, including the scan range (e.g., whole‐body vs. vertex to thighs) should be pre‐established before the patient arrives at the facility and should be written in this technologist's worksheet.

Use of these forms should be used to ensure that the tests from Table 6 and described below are correctly performed.

**Table:** 

PS1	The name, date of birth, and other medical information should be checked with the patient prior to beginning any procedure. At least two extra patient provided information should match the medical requisition.^30^
PS2	Refer to the PET/CT technologist's form and check that the imaging protocol and patient preparation conform to requisition. If the patient is unable to comply, special accommodations may be required. If ambiguity exists, consult with the reporting physicians and/or on service referring physician.^30^
PS3	Ensure that patient positioning conforms to treatment plan including use of all immobilization devices and appropriate position indexing. These parameters should be available in the technologist's worksheet form. Perform the scout/topogram acquisition and ensure that positioning and FOV are set as defined in the technologist's worksheet before continuing the PET/CT acquisition.^10^
PS4	Image acquisition parameters should be preconfigured on the acquisition system for all common procedures and documented in a clinical protocol. The scanning protocol should be written in advance in the technologist's worksheet. Before performing the scout acquisition, the selection of the protocol should be checked to match what is specified in the technologist's form. If deviations from preconfigured protocols are required, these should be documented by the technologists.^30^
PS5	Accurate registration between PET and CT scans must be ensured during PET image reconstruction. If significant patient motion has occurred between scans (especially at targets of interest) repetition of scans on a limited FOV should be considered. Rigid motion correction can be considered if appropriate, but small corrections are discouraged, as they typically exacerbate misregistration. This can be tested by generating fused images of PET and CT and visually inspecting for mismatch artifacts. The patient should not be removed from the scanner until the images have been checked to avoid having to obtain an extra CT following the ALARA principle.^30^
PS6	Global image quality QC tests include checking for artifacts, verifying FOV coverage, appropriate CT contrast (if applicable), and that correct series description names have been set on the images so that nuclear medicine physicians can easily understand what they are looking at.^30^

### 4.1 Ancillary equipment

PET images are typically reported as standard uptake values (SUV) that have been shown to be accurate within ±10% across a wide range of scanner models with appropriate QA and method standardization.^31^ Since SUV is calculated using patient weight, periodic QA should be carried out on patient weight scales in accordance with the vendor's recommendations.^10^ Accuracy and precision on the order of ±1 kg corresponds to 1%–2% patient weight error and is on par with clinical sources of variability including patient clothing, bowel content, and hydration state, but ±0.2 kg is readily achievable with clinical devices and routine QC. Likewise, PET images are often scaled to standard uptake based on lean‐body mass (SUL), which are computed based on patient height. Height measurement apparatuses should be accurate to within 5 mm.

Dose calibrators are used to measure the patient‐administered activities which factors into the SUV calculation, and they serve as a reference for calibration of the PET system. Therefore, they must undergo routine QC to ensure consistency. If multiple dose calibrators are in use, cross calibration must also be ensured. Vendor recommendations should be followed, while professional society guidelines also layout periodic QC including consistency, accuracy, linearity, and geometric and positioning sensitivity testing.^24^

Synchronization of clocks between dose calibrators and PET imaging devices is required for accurate radionuclide decay correction and may be aided by automated device clock synchronization with a centralized time server. Regardless, daily QC of dose calibrator and PET times is recommended, with an emphasis when adjusting to daylight savings times.

### 4.2 Image transfer and compatibility

Widespread adoption of DICOM standards for image and RTP transfer has aided compatibility between imaging, diagnostic visualization, and treatment planning systems. Target volumes can therefore be delineated on either diagnostic imaging or treatment planning workstations, depending on the preferred tools and workflow. Nevertheless, the commissioning of new systems should entail the validation of proper data transfer including specific emphasis on image orientation, pixel size, spatial positioning offsets, and image unit scaling (e.g., SUV). Validations should replicate the clinical workflow and can utilize phantom scans or a patient scan augmented with physical markers that are visible in the image. Marker locations, sizes, and separation can be measured in images and validated against the empirical setup. Suitable markers include:

1. Radioactive point sources (e.g., ^22^Na)
2. Radioactive dilution standards (e.g., sealed vials with known dilutions of FDG) for validating activity quantification.
3. Thin metal wires that are visible on CT, but do not introduce artifacts.

Commissioning acceptance testing and routine QC testing for RTP systems are detailed in the AAPM TG‐53 report^32^ and in the IAEA Technical document N. 1583.^18^

### 4.3 Image reconstruction and processing

Image reconstruction and processing parameters can influence image characteristics including target to background uptake ratio, spatial resolution, and noise. These in turn may influence the perceived target size and intensity. For consistent volume delineation, image reconstruction and processing methodologies should be carefully derived, validated, and preserved. As patients may be imaged on different scanners during the course of their treatment, a need to harmonize image reconstruction and processing across the patient catchment region is ideal, especially for quantitative assessment of tumor response to treatments including RT. Changes to image reconstruction and processing methodology should be coordinated between the imaging and radiation therapy teams. Likewise, the use of institutionally standardized default image display parameters (e.g., color maps, window/level, and image fusion level) is recommended.

### 4.4 Deviations from these guidelines

This recommendation document details a rigorous set of tests and prescribed schedules for a general audience. Adaptations may be made to enhance feasibility and efficiency in local practice and specific scenarios. Adaptations should be made by subject matter experts familiar with the equipment, clinical application, and work environment. For example, fluctuations in scanner sensitivity can be monitored by the quarterly calibration factor measurements, which could make annual repetition of the commissioning tests optional.

Likewise, centers acquiring dual energy CT (DECT) on their systems should consider the implementations of DECT‐specific quality‐control tests as recommended in recent articles.^33^, ^34^ In both articles a QC protocol is described using a DECT specific phantom with a known quantity of iodine which is quantified via DECT and the difference is evaluated. Both articles recommend the DECT test be performed weekly, however, Nute et al.^33^ note that monthly testing may be acceptable if weekly testing is not feasible. In the study by Nute et al., the tolerances for the DECT tests are recommended to be both a fixed error of ±1 mg/mL and a percentage error of 10%. While a study conducted by Green et al., did not set specific tolerances, they did note that there should be low variability in terms of iodine density.^34^

### 4.5 New technologies

With advancement in instrumentation and automated fault testing, some tests may become obsolete (e.g., annual x‐ray generation); in‐depth understanding of the technology and consultation with the vendor are recommended prior to relaxing QC activities. On the other hand, with the increased application of automation, optimization, and artificial intelligence (AI) into every aspect of medical imaging and RTP, PET/CT for RT may be especially prone to generating harms due to unexpected behaviors from these systems. As AI enters the clinic, the community will need to develop new quality assurance that targets these vulnerabilities to ensure that systems are safe, accurate and predictable. The reader is referred to the work done by Vandewinckele et al.^35^ for an overview and recommendations for QA of AI in radiotherapy including image automated segmentation, automated treatment planning, and synthetic CT.

### Clinical practices

Often the quality of clinical data is limited not by the imaging equipment, but rather by clinical factors. Appendix A summarizes these factors and makes recommendations towards enhanced clinical quality.

## 5 CONCLUSION

Quality assurance of PET/CT for RTP requires additional efforts to diagnostic PET/CT alone and collaboration between team members. Nevertheless, the incremental effort is relatively small compared to the potential clinical benefit, and is therefore a feasible undertaking.

## AUTHOR CONTRIBUTIONS

Ran Klein, PhD: Primary author, corresponding author; Mike Oliver, PhD, MCCPM: Contributing author, proofing; Dan La Russa, PhD, FCCPM: Contributing author, proofing; John Agapito, MS, MCCPM: Contributing author, proofing; Stewart Gaede, PhD, MCCPM: Contributing author, proofing; Jean‐Pierre Bissonnette, PhD, MCCPM, FCOMP: Contributing author, proofing, initiator; Arman Rahmim, PhD, DABSNM: Contributing author, proofing, coordination with COMP; Carlos Uribe, PhD, MCCPM: Secondary author

## DISCLOSURES

Ran Klein receives revenue shares from the sale of Rubidium generators from Jubilant‐DraxImage and from the sales of myocardial flow quantification software from Invia Medical Solutions. None of the authors have conflicts of interest to disclose related directly to this work.

## DISCLAIMER

All information contained in this document is intended to be used at the discretion of each individual centre to help guide quality and safety program improvement. There are no legal standards supporting this document; specific federal or provincial regulations and license conditions take precedence over the content of this document. As a living document, the information contained within this document is subject to change at any time without notice. In no event shall the Canadian Partnership for Quality Radiotherapy (CPQR) or its partner associations, the Canadian Association of Radiation Oncology (CARO), the Canadian Organization of Medical Physicists (COMP), and the Canadian Association of Medical Radiation Technologists (CAMRT), be liable for any damages, losses, expenses, or costs whatsoever arising in connection with the use of this document.

## APPENDIX A CLINICAL QUALITY

### Patient preparation

Specific patient preparation consideration should be given depending on the PET tracer, disease state of patient, and clinical task. Specific guidelines for tracers and indications are continuously being developed by professional bodies. The Society of Nuclear Medicine and Molecular Imaging (SNMMI) and the European Association of Nuclear Medicine (EANM) commonly publish joint guidelines that are freely available through their respective websites, including for FDG^30^, ^37^ and ^68^Ga‐PSMA.^38^

For accurate SUV/SUL scaling, patient weight and height should be measured with a high‐quality scale. In addition, the activity administered to the patient must be accurately measured, including the residual activity in the syringe after injection as well as time of injection (for radioactive decay correction).

### CT Contrast Agent

The use of CT contrast agents is commonly applied for improved organ delineation in RTP. Concerns regarding suboptimal attenuation correction from contrast CT have been largely addressed except for cases of high concentration (e.g., arterial phase).^30^, ^37^ Venous phase and delayed enhancement CT‐contrast imaging may produce small changes in SUV.^39^ Nevertheless, with the added information of PET for RTP, the need for CT contrast may be reduced. At the expense of extra scan time and radiation exposure to the patient, two CT scans may also be obtained: without and with contrast.

### Patient Positioning

Utilization of diagnostic PET for RT simulation is typically ill‐advised due to differences in patient positioning between imaging and therapy sessions. PET acquisition on a flat tabletop and with appropriate immobilization devices is preferable as this enables better software‐based registration between the PET and simulation CTs.

Ideally RTP and simulation should be performed using hardware registered PET and CT (i.e., hybrid systems), and with appropriate patient positioning by a qualified radiation therapist. The use of fiducial markers, a flat bed, patient immobilization devices, and dedicated laser alignment hardware should be integrated into the PET process for optimal registration with RT delivery devices. The PET/CT patient positioning should replicate that of RT as nearly as possible using identical apparatuses.

### PET/CT registration

For accurate attenuation correction and SUV quantification, it is assumed that hardware registration between PET and CT is sufficient. Nevertheless, in the presence of patient motion this assumption may be violated (typically regionally). PET/CT registration QA should be performed in every case prior to patient removal from the PET imaging bed, as is common practice in diagnostic imaging.^37^ Repeat imaging of body regions in which gross misregistration is apparent may be undertaken as required. Manual alignment may be appropriate, but adjustment of small misregistration is not recommended as it may introduce errors due to human factors.

PET and CT misregistration in the lung and liver regions is unavoidable due to the long imaging time of PET (2‐3 min per bed position) versus that of CT. Normal breath‐hold techniques during the CT acquisition are recommended,^37^ but the use of 4D CT should be considered in cases where accurate target delineation in respiratory‐motion‐affected regions is vital.

### Respiratory Motion

Gated PET (4D) is recommended to account for reciprocating organ and target motion in lung, heart, diaphragm, and upper abdominal regions.^8^, ^40^, ^41^ In conjunction with appropriate therapy delivery equipment, target tracking and/or dose rate modulation can be used to deliver more accurate and conformal dose distributions. Although new data driven or device‐less methods that estimate the respiratory wave function using the projection data of PET are being introduced into clinical systems, gated PET typically relies on external respiratory triggering hardware (e.g., optical tracking or pressure belt) to assign detected events to corresponding phases in the respiratory cycle. Respiratory equipment at the delivery unit may differ and may not provide identical information regarding the magnitude of motion. Gated PET in most scanners currently in use still relies on external respiratory equipment and specific QA is required to ensure adequate correlation between gating systems for optimal dose delivery.

With list‐mode data acquisition being a standard feature of modern PET systems, PET reconstruction of static (3D) and respiratory gated (4D) images is possible from a single PET acquisition. To compensate for lower count‐statistics per gate, however, it may be desirable to acquire motion effected body regions with longer time per bed‐stop, especially in the presence of small, low intensity targets. Moving objects are blurred in static images, typically making lung lesions appear fainter and larger, but motion correction software is becoming increasingly available to reconstruct motion frozen PET while preserving 100% of the data.

While other types of motion, such as cardiac contraction, gross patient motion, and organ creep are measurable, they are largely ignored in the context of RT.

### Time to Therapy

Due to the dynamic nature of cancer, the time between diagnostic and/or pre‐treatment imaging and delivery of therapy may be a critical factor for accurate target delineation. Geiger et al.^42^ and Everitt et al.^43^ demonstrated that in non‐small‐cell lung cancer over the course of even a few weeks, a significant number of patient were upstaged due to increases in tumor FDG avidity, tumor size, number of nodes, and metastatic state. These changes in staging influenced the intent to treat from curative to palliative within several weeks and are consistent with previous findings in both lung and other cancers.^42^ Hence, the clinical workflow should target RT delivery within 2 weeks of PET/CT for RTP.

### Protocols

Patient mispositioning, inaccurate communication, and operator error remain large sources of variability in RTP and can be mitigated using clear, predefined protocols. Protocols should be body site specific and should contain instructions regarding patient positioning, immobilization devices, setup instructions, image acquisition protocols and parameters, scan limits, use of contrast agents, and any additional special instructions.^28^ Image acquisition parameters should be preconfigured as imaging protocols on the modality workstation to reduce errors due to human factors and improve workflow. Likewise, contrast and/or tracer injection systems should be preconfigured.

### Nomenclature

Because PET/CT for RTP involves multidisciplinary interactions, it is especially important that effective communication be facilitated using standardized nomenclature such as proposed in the AAPM TG‐263 report.^44^ Standardized nomenclature may incrementally benefit multicenter clinical trials and development of artificial intelligence‐based applications.^45^

### Overall System Test

Integration of all the components in an RT workflow should be tested with a system level validation test whenever changes are made to equipment, software, or workflow. System tests should use a validation phantom and a typical clinical workflow to test object alignment and orientation, image acquisition, image transfer, image processing, treatment planning, transfer of plan to therapy device, treatment delivery verification including image guidance and creation of documents. Delivery of the desired radiation plan may be validated using dosimetry equipment but is beyond the scope of this document.

### Roles and Responsibilities

Even when the intention of a PET/CT study is for RTP, best practice is that a nuclear medicine and radiology trained physician reviews the study in a timely manner to evaluate disease progression and to detect incidental findings.

Quality assurance is an institutional responsibility and therefore requires the collaboration of all care providers and support staff. Physicists are charged with ensuring optimal functioning of instrumentation and software but are rarely present during the immediate course of clinical care. Technologists are often the first to witness anomalies, whether it is patient compliance, equipment failure, or inappropriate requisitions. It is vital that technologists are empowered to resolve errors when appropriate and to freely communicate concerns and observations within the circle of care. Imaging physicians and radiation oncologists routinely view image and other clinical data and are therefore well positioned to identify errors and artifacts in a timely manner. Thus, they should be well trained to identify these anomalies and to draw attention to them in a timely manner. The biomedical engineering team is charged with ensuring that maintenance is performed to the highest standard and in coordination with the manufacturer's guidelines. Finally, the management team is essential to emphasizing the value of quality and supporting it with adequate resources.

Reference^46^ is a good resource that presents different image artifacts and discusses possible causes.

### Comparative Studies

As with any comparative study, it is assumed that patient preparation, image acquisition, and image reconstruction parameters are well controlled. Nevertheless, previously published multicenter clinical trials have demonstrated that compliance with professional guidelines may be low and could introduce undesired variability to the study data.^47^ Likewise, for studies with baseline and follow‐up scans, it is pertinent to ensure that both scans are acquired under similar, pre‐defined conditions. Special considerations must be given if images originate from two different PET/CT systems, as harmonization across devices, especially by different vendor/models, may not be achievable. Pre‐study qualifying scans^48^ and routine quality control over the course of a research study are pertinent to ensuring high quality data. Much of the required data (e.g., image acquisition and reconstruction parameters, tracer uptake times, blood glucose level) may be available from the image DICOM header, but care should be taken to ensure that these data are not stripped during data anonymization, transfer, and conversion. Other data should be captured in clinical report forms (CRF) and checked for quality. Rapid feedback and guidance of imaging sites by the core lab is essential to achieving and maintaining optimal data quality throughout the course of the study.

Multicenter trials may especially benefit from the use of a standardized phantom which facilitate qualitative and quantitative validation of image quality against known activity distributions and enables objective comparison between sites and equipment. Such initiatives have been well demonstrated by professional groups including the Society of Nuclear Medicine and Molecular Imaging (SNMMI) Clinical Trials Network,^49^ American College of Radiology Nuclear Medicine Accreditation Program,^50^ EANM Research Ltd (EARL),^51^ and Ontario Clinical Oncology Group (OCOG),^52^ and therefore phantom data may be readily available at active research and/or accredited sites. Traditionally, these phantoms focus on PET uptake quantification and image quality, but for RTP an emphasis should also be placed on aspects of target volume delineation including location and size.
